# Towards efficient solutions: A novel approach to quadratic nonlinearity in boundary value problems

**DOI:** 10.1371/journal.pone.0317752

**Published:** 2025-05-23

**Authors:** Salima Kouser, Shafiq Ur Rehman, Yasser Elmasry, Waqar Azeem Khan, Fayyaz Ahmad, Hamza Khan

**Affiliations:** 1 Department of Mathematics, University of Engineering and Technology, Lahore, Pakistan; 2 Department of Mathematics - College of Science - King Khalid University, Abha, Saudi Arabia; 3 Department of Mathematics, Mohi-ud-Din Islamic University, Nerian Sharif, Azad Jammu and Kashmir, Pakistan; 4 Department of Applied Sciences, National Textile University, Faisalabad, Pakistan; Vignan’s Institute of Information Technology, INDIA

## Abstract

The Newton method is a classical method for solving systems of nonlinear equations and offers quadratic convergence. The order of convergence of the Newton method is optimal as it requires one evaluation for the system of nonlinear equations and the second for the Jacobian. Many boundary value problems in nature have quadratic non-linearity and the corresponding system of nonlinear equations associated with their discrete formulation has constant 2^*nd*^-order Fréchet derivatives. We try to get benefit from this information and develop a single-point iterative method to solve such a system of nonlinear equations with quadratic nonlinearity. In our proposed single-point iterative method, we perform one evaluation of a system of nonlinear equations and another for Jacobian. In total, there are two functional evaluations, and we do not count the evaluation of the 2^*nd*^-order Fréchet derivative as it is constant in all the iterations of the method. The convergence order (CO) of our proposed method is four. The efficiency index of our method is 4^1/2^ = 2 which is higher than that of the Newton method 2^1/2^ = 1.4142. To quantify the functionality of our proposed algorithm, we have performed extensive numerical testing on a collection of test problems with quadratic nonlinearity.

## 1 Introduction

The numerical solution of a nonlinear equation in its closed form is not always possible, and hence numerical iterative schemes provide us with an alternative way to find them. The exact method, for instance, the quadratic formula to obtain the roots of a quadratic equation, is an example of the direct method. However, when dealing with a system of nonlinear equations (SNLEs), the situation becomes more complicated in terms of determining the exact empirical formulae. The practical way to find the numerical solution of such SNLEs is through numerical iterative methods. In many ways, these numerical methods differ from exact numerical methods or exact symbolic methods. The iterative methods always require an initial guess to start the iterative procedure, but on the other hand, the exact methods do not require any initial guess. The total number of binary operations can be counted for the exact method, while in the case of iterative methods, we can have an estimate of the computational cost. The iterative methods need some stopping criteria.

The symbolic exact methods may suffer from numerical instabilities when we substitute numerical values in place of symbols. The stable numerical algorithms avoid numerical instabilities.

The bisection method, the regula-falsi method, the Newton method [1], and the secant method are all methods for the numerical approximation of nonlinear equations. The bisection and false-position methods are closed-form methods because they require the knowledge of an interval containing the root. On the other hand, the Newton and Secant methods are open methods because we need a single initial guess to start the iterative process. The generalisation of bisection and regula-falsi for the SNLEs is a bit hard and may be possible in some sense. The open methods, for instance, the Newton and Secant methods, are excellent candidates for multidimensional generalization.

The root(s) of a nonlinear equation or SNLEs can be classified into two categories, namely, simple roots and roots with multiplicity. Let $x=[x_{1},x_{2},\cdots ,x_{n}{]}^{T}\in \mathbb{R}$ be a multi-dimensional vector and F(x)=0, where $F:D\subset {\mathbb{R}}^{n}\to {\mathbb{R}}^{n}$ represents the SNLEs. We also assume that F is sufficiently smooth, that is, that all of its Fréchet derivatives exit up to some suitable order. If the limit [2]


$$\lim_{h\to 0}\frac{\left||F(x+h)-F(h)-A\;h|\right|}{\left||h|\right|}=0\;,$$

exits, then the first order Fréchet derivative AofFexistsatx. Whereas, the higher-order derivatives are computed by following the recursion


F(x)=Jcobian(F(x)),

$$F^{\left(i\right)}(x)w^{\left(i-1\right)}=\text{Jcobian}(F^{\left(i-1\right)}(x)z^{\left(i-1\right)})\;\;i\geq 2\;,$$

where w and z are not the functions of x and


$$F^{\left(3\right)}w^{3}=\text{Jacobian}((\text{Jacobian}(\text{Jacobian}(F(x))w))w)w\;.$$

It is worth mentioning that the Fréchet differentiable function could be linearized in the neighborhood of a give point x if $F^{\prime }(x)$ exists. From the linearization of F, we can derive the Newton method. Before proceeding, we define the various types of roots. A vector α is called the root of F(x)=0 if


F(α)=0.

The root α is simple if


$$\text{det}(F^{\prime }(\alpha ))\neq 0\;,$$

that is, if the Jacobian of F at α is non-singular. Otherwise, the root α is not a simple root. In this study, we assume that the root α is a simple root in all cases. Let $x_{n}$ be the nth approximation of α, and we want to get the new approximation because it may reduce the norm of SNLEs. If $x_{n}$ is the approximation of α, then


$$x_{n}\approx \alpha \;,$$

and we are looking for h, such that,


$$x_{n}+h=\alpha \;,$$

where h is a correction vector. As discussed earlier, the Fréchet differentiability helps in the linear approximation of SNLEs, so we have


$$F(\alpha )=F(x_{n}+h)=0,$$

$$F(x_{n}+h)\approx F(x_{n})+h\;F^{\prime }(x_{n}),$$

$$F(x_{n})+h\;F^{\prime }(x_{n})\approx 0,$$

$$h\approx -F^{\prime }(x_{n}{)}^{-1}\;F(x_{n})\;.$$

This yields an approximation of the correction vector h and a new approximation of α


$$\alpha =x_{n}+h\approx x_{n}-F^{\prime }(x_{n}{)}^{-1}\;F(x_{n})\;.$$

It could also be written as


$$x_{n+1}=x_{n}-F^{\prime }(x_{n}{)}^{-1}\;F(x_{n})\;.$$

The above formula is called the Newton method. In practice, we never compute the inverse of the Jacobian matrix; instead, we solve systems of linear equations. The practical way to write the Newton method is


$$\text{Newton method}=\left\{ \begin{array}{cc} x_{n} & n\text{th approximation to the root }\text{ɑ}\}\\ F^{\prime }(x_{n})h=F(x_{n}) & \text{solve the system of linear equations}\\ x_{n+1}=x_{n}-h & \text{compute the (n+1) th approximation of the root. } \end{array}\right.$$

It’s worth noting that the Newton method has two function evaluations F, $F^{\prime }$, as well as a single system of linear equations solution for a single iteration. The number *m* is the CO of numerical iterative method without memory if


$$e_{n+1}\propto e_{n}^{m}\;,$$

where, $e_{n}=x_{n}\;-\;\alpha$ denotes error at the *n*th-step. Since α is unknown to us, it is very difficult to get the exact value of *m* by using the above definition. In practice, we computationally approximate the order of convergence as


$$\text{COC}=\frac{\log\left(||F(x_{n+2})||/||F(x_{n+1})||\right)}{\log\left(||F(x_{n+1})||/||F(x_{n})||\right)}\;.$$

The numerical iterative methods are classified into different classes. We call an iterative method a multi-point method [4, 5, 8–12] if the function and derivative evaluations are performed at different points in a single iteration. If all the functional evaluations are concentrated at a single point, then such a numerical method is called a single-point iterative method. Usually, the higher-order methods are multi-point and the lower order methods are single-point iterative methods. The multi-point methods are more efficient compared to single-point iterative methods when the CO is high. Another classification is single-step and multi-step methods [3, 10–12]. The computation of the Jacobian can be expensive. To avoid the new computation of Jacobian and its LU-factors, it is desirable to freeze Jacobian in a single iteration and develop a strategy to attain a high CO. A Newton multi-step and multi-point iterative method [3] could be formulated as


$$\text{Multi-step and multi-point Newton method}=\left\{ \begin{array}{cc} & x_{1}=x_{0}-F^{\prime }(x_{0}{)}^{-1}\;F(x_{0})\\ & \text{for i from 1 to s-1 do}\\ & \;1.5emx_{i+1}=x_{i}-F^{\prime }(x_{0}{)}^{-1}\;F(x_{i})\\ & \text{end do}\;. \end{array}\right.$$

One can observe that the Jacobian is fixed and only function evaluations are performed. There is a single Jacobian evaluation and *s*-function evaluations. The CO of this method is s+1. In the first step, we have the Newton method, which has a quadratic CO, and for each step of the loop, we increase the previous step order by 1, as the length of the loop is *s*−1. As a result, the CO is 2 + *s*−1 = *s* + 1. When solving a system of linear equations at each step, it is preferable to find the LU-factors for the frozen Jacobian $F^{\prime }(x_{0})$ if possible. The corresponding lower and upper triangular systems are solved by backward and forward substitutions, which makes the overall computational cost economical.

In recent years, many researchers have contributed to developing numerical iterative schemes for SNLEs. A parameterized multi-point and multi-step method is proposed in [3]. The authors of [4] constructed a multi-step iterative method using Jacobian information at two different points. The discretization of boundary and initial value problems gave rise to SNLEs, which can be of a special type due to structure differential equations. Some methods in this direction are proposed in [5, 6]. For nonlinear equations, the optimality of the CO is well defined for the numerical iterative method, but such a notion is not well defined for the SNLEs. For instance, for a single nonlinear equation, the Newton method has quadratic convergence, and this CO is optimal according to the Kung-Traub’s (KT) conjecture [1]. According to the KT conjecture, if a numerical iterative method without memory has *r*-functional evaluations in a single stance of the method, then the optimal CO is


$$\text{optimal convergence order}=2^{r-1}\;.$$

There is no KT-conjecture for iterative methods of solving SNLEs. Generally, if there are two functional evaluations, then the optimal CO is two. Similarly, if there are three functional evaluations, then the optimal order is four. In this context, an optimal order method of order four is proposed. In [11], another multi-step iterative method is proposed.

The development of numerical methods using a 2^*nd*^-order Fréchet derivative is not practical because the computational cost of a 2^*nd*^-order Fréchet derivative is very high as it is a tensor of rank three. But it is not the case when we focus on SNLEs associated with boundary value problems (BVPs). Consider a two-point 2^*nd*^–order BVP


$$y^{\prime \prime }(x)+f(y(x))=g(x)\;,\;x\in (a,b)\;,\;y(a)=y_{a}\;,\;y(b)=y_{b}\;$$

where f(·) is a nonlinear function. After discretization, we get a SNLEs

F(y)=Ay+f(y)−g=0, (1)

where $y=[y(x_{1}),y(x_{2}),\cdots ,y(x_{n})]$, $y_{0}=y_{a}$, and $y_{n+1}=y_{b}$. Here A is the functional matrix for the 2^*nd*^ order derivative and $\left\{x_{1},x_{2},\cdots ,x_{n}\right\}$ is a partition of the interval [a,b] and the order vectors $g=[g(x_{1}),g(x_{2}),\cdots ,g(x_{n}){]}^{T}$ and $f(y)=[f(y_{1}),f(y_{2}),\cdots ,f(y_{n}){]}^{T}$. Consider that we have included the boundary conditions in (1). The Fréchet derivatives of (1) can be obtained as


$$F^{\prime }(y)=A+\text{diag}(f^{\prime }(y))$$

$$F^{\prime \prime }(y)=\text{diag}(f^{\prime \prime }(y))\;,$$

where diag stands for the diagonal matrix. The $F^{\prime \prime }$ is a tensor of rank three. To convert it to a tensor of rank one, which is a vector, we need to multiply it by two tensors of rank one, i.e.,


$$F^{\prime \prime }(y)w=\text{diag}(f^{\prime \prime }(y)⊙w)=\text{matrix}\;,$$

$$F^{\prime \prime }(y)w\;z=f^{\prime \prime }(y)⊙w⊙z=\text{vector}\;,$$

where ⊙ is element-wise multiplication operation. We can see that in the case of BVPs, the computation of 2^*nd*^ order Fréchet derivative could be simple and computationally economical. By getting motivation from the economical perspective of a higher-order Fréchet derivative (where it is possible) for the discretized BVPs, we propose a single-point numerical iterative method. We concentrate on the case where the underlying nonlinearity is quadratic; for example, our method is valid when f(y) in (1) is $f(y)=y^{2}$. Now, we describe our single-point numerical iterative method for SNLEs


F(x)=0

under the condition that


$$F^{\prime \prime }(x)=\text{constant tensor of rank 3}$$

and


$$F^{\left(i\right)}(x)=\text{zeros tensor of rank i+1}\;,\;i\geq 3\;.$$

$$\text{Proposed method}=\left\{ \begin{array}{cc} x_{n} & n\text{th approximation }\\ F^{\prime }(x_{n})\phi_{1}=F(x_{n}) & \phi_{1}\text{ tensor of rank 1}\\ M=F^{\prime \prime }\;\phi_{1} & M\text{ tensor of rank 2}\\ F^{\prime }(x_{n})\phi_{2}=M\;\phi_{1} & \phi_{2}\text{ tensor of rank 1}\\ F^{\prime }(x_{n})\phi_{3}=M\;\phi_{2} & \phi_{3}\text{ tensor of rank 1}\\ x_{n+1}=x_{n}-\phi_{1}-\frac{1}{2}\;(\phi_{2}+\phi_{3})\;, \end{array}\right.$$ (2)

$F^{\prime \prime }$ is a constant tensor of rank 3 and all higher order tensor of rank *i* are zeros tensors, i.e., $F^{\left(i\right)}=0\text{\textasciitilde{}for\textasciitilde{}}i\geq 3$. The CO of our proposed method (2) is four and the corresponding error equation is


$$e_{n+1}=5\;{\left(\frac{F^{\prime }(\alpha {)}^{-1}\;F^{\prime \prime }}{2}\right)}^{3}\;e_{n}^{4}\;.$$

There is one evaluation of SNLEs $F(x_{n})$ and one evaluation of Jacobian $F^{\prime }(x_{n})$, so in total we have two functional evaluations. We do not count the evaluation of $F^{\prime \prime }$ as it is a constant tensor of rank 3. Two matrix-vector multiplications, one scalar-vector multiplication, one LU decomposition of $F^{\prime }(x_{n})$, and three lower and upper triangular system solutions are available. Whereas the computational cost of M depends on the structure of quadratic nonlinearity.

## 2 Convergence analysis

For convergence analysis, we define


$$C_{r}=\frac{1}{r!}F^{\prime }(\alpha {)}^{-1}F^{\left(r\right)}(\alpha )$$

for $r\geq 2\text{\textasciitilde{}and\textasciitilde{}}C_{1}=F^{\prime }(\alpha )$. Using the Taylor’s expansion, we can expand the $F(x_{n})\;\text{around}\text{the\textasciitilde{}}\alpha$

$$F(x_{n})=\;F(x_{n}-\alpha +\alpha )=F(e_{n}+\alpha )\;=\;F(\alpha )+F^{\prime }(\alpha )e_{n}+1/2\;F^{\prime \prime }(\alpha )e_{n}^{2}\;=\;F^{\prime }(\alpha )e_{n}+1/2\;F^{\prime \prime }(\alpha )e_{n}^{2}\;=\;F^{\prime }(\alpha )(e_{n}+1/2\;F^{\prime }(\alpha {)}^{-1}F^{\prime \prime }(\alpha )e_{n}^{2})\;=\;C_{1}(e_{n}+C_{2}e_{n}^{2})\;,$$ (3)

because α is a root, F(α)=0. The higher derivatives of $F(x_{n})$ are

$$F^{\prime }(x_{n})=C_{1}(1+2\;C_{2}e_{n})\;,\;F^{\prime \prime }(x_{n})=2\;C_{1}\;C_{2}\;,\;F^{\left(i\right)}(x_{n})=0\;,\;i\geq 3\;.$$ (4)

**Theorem 1.** *Suppose that F is at least twice Fréchet differentiable in the nonempty open convex domain *D* and initial guess $x_{0}$ is sufficiently close to α. Then, $\{x_{n}{\}}_{n\geq 0}$ converge to α with at least four CO.*

**Proof 2.** *To compute the value of $\phi_{1}$, we need to compute the Taylor’s expansion of $F^{\prime -1}(\alpha )$ as*

$$F^{\prime -1}(\alpha )=(1-2\;C_{2}\;e+4\;C_{2}^{2}e_{n}^{2}\;-8\;C_{2}^{3}e_{n}^{3}\;+16\;C_{2}^{4}e_{n}^{4}\;)\;C_{1}^{-1}\;.$$ (5)

*The value of $\phi_{1}$ is*

$$\phi_{1}=e_{n}-C_{2}e_{n}^{2}\;+2\;C_{2}^{2}e_{n}^{3}\;-4\;C_{2}^{3}e_{n}^{4}\;+8\;C_{2}^{4}e_{n}^{5}\;+16\;C_{2}^{5}e_{n}^{6}\;.$$ (6)

*Similarly, the values of $\phi_{2}\text{\textasciitilde{}and\textasciitilde{}}\phi_{3}$ are*

$$\phi_{2}=2\;C_{2}e_{n}^{2}\;-8\;C_{2}^{2}e_{n}^{3}\;+26\;C_{2}^{3}e_{n}^{4}\;,$$ (7)

$$\phi_{3}=4\;C_{2}^{2}e_{n}^{3}\;-28\;C_{2}^{3}e_{n}^{4}\;.$$ (8)

*Substituting these values in*


$$e_{n+1}=e_{n}-\phi_{1}-\frac{1}{2}(\phi_{2}+\phi_{3})\;.$$

*we get the error equation*


$$e_{n+1}=5\;C_{2}^{3}\;e_{n}^{4}\;.$$

*Which shows that the CO of our proposed algorithm is four.*

## 3 Numerical simulations

The implementation of our proposed method requires the discretization of BVPs, and we have adopted the Chebyshev pseudospectral method [7, 8] to get the operational matrices for the differentiation of different orders.

### 3.1 System of nonlinear equations in 2-D

To address the correctness of the order of convergence of our proposed algorithm, we solve a set of problems and compute the COC.

**Table:** 

System of nonlinear equations: 1	System of nonlinear equations: 2
(line-circle)	(circle-circle)
$x_{1}^{2}+x_{2}^{2}-1=0$,	$(x1-0.5)2+(x_{2}-0.5)2-1=0$,
$x_{1}+x_{2}-0.5=0$,	$x_{1}^{2}+x_{2}^{2}-1.5=0$,

System of nonlinear equations: 3	System of nonlinear equations: 4
(circle-parabola)	(circle-hyperbola)
$x_{1}^{2}+x_{2}^{2}-1=0,$	$x_{1}^{2}+x_{2}^{2}-2=0$,
$-4x_{1}^{2}+x_{2}=0\;.$	$x_{1}^{2}-x_{2}^{2}-1=0$ .

System of nonlinear equations: 5
(4-D nonlinear system)
$x_{2}x_{3}+x_{4}(x_{2}+x_{3})=0$
$x_{1}x_{3}+x_{4}(x_{1}+x_{3})=0$
$x_{1}x_{2}+x_{4}(x_{1}+x_{2})=0$
$x_{1}x_{2}+x_{3}(x_{2}+x_{4})=1.\;.$

The aforementioned five systems of nonlinear equations have quadratic nonlinearity. The proposed numerical iterative method is used to solve these five problems. The convergence analysis for multiple geometric configurations commences with distinct initial guesses for each setup. For the line-circle interaction, the initial guess is set at [0.5,2.0], indicating an exploratory starting point in the two-dimensional space. Similarly, the circle-circle system begins with an initial guess of [0.5,0.1], slightly offset within the plane. The circle-parabola configuration introduces a more varied starting position with an initial guess of [−0.4,0.4], suggesting a potential intersection or tangent solution. The circle-hyperbola scenario is approached with an initial guess of [1.0,0.4], positioning the search in a region of potential interest. Lastly, the 4-D nonlinear system, which encapsulates a complex dynamic interaction, starts from a multi-dimensional guess of [0.5,0.5,0.5,0.5,], evenly distributing the initial probing across its four dimensions. These initial guesses are pivotal for the iterative methods employed in seeking the solutions, illustrating the tailored approach based on the geometric nature and dimensionality of each system. The computational orders of convergence are reported in Table 1. It is evident that the computational orders of convergence agree with the claimed OC of the proposed numerical iterative method (2).

**Table 1:** The nonlinear equation system’s norms are shown, and the COC is bolded and enclosed in parenthesis.

Line-Circle	Circle-Ellipse	Circle-Parabola	Circle-Hyperbola	4-D Nonlinear system
3.2	7.5e1	6.8e-1	8.4e-1	6.2e-1
1.7	1.1e2	1.0	2.1e-1	1.8e-1
4.4e-2 (5.7)	1.9e1 (-0.47)	1.1e-2 (-11.6)	4.1e-4 (4.5)	5.4e-5 (6.6)
3.8e-7 (3.3)	8.0e-1 (1.1)	5.2e-9 (3.2)	1.8e-14 (3.8)	6.5e-19 (3.9)
2.6e-27 (3.8)	6.1e-3 (1.9)	9.9e-35 (4.1)	6.7e-56 (4.0)	1.4e-74 (4.0)
5.0e-108 (4.1)	1.1e-10 (3.7)			
	1.1e-41 (3.9)			
	1.4e-165 (4.1)			

The numerically computed solutions as follows: For the line-circle configuration, the solution is *x*_1_ = −0.4114 and *x*_2_ = 0.9114. In the case of the circle-circle interaction, the solution is given by *x*_1_ = −0.2071 and *x*_2_ = 1.2071. For the circle-parabola system, the computed values are *x*_1_ = −0.4698 and *x*_2_ = 0.8828. The circle-hyperbola configuration results in *x*_1_ = 1.2247 and *x*_2_ = 0.7071. Finally, the 4-D nonlinear system presents a more complex solution set with *x*_1_ = 0.81650, *x*_2_ = 0.81650, *x*_3_ = 0.81650, and *x*_4_ = −0.40825, illustrating the intricate dynamics involved in higher-dimensional systems.

One may check the second order Fréchet derivatives of SNLEs with quadratic nonlinearity is constant. Suppose, if $v_{1}=[h_{1},h_{2},h_{3},h_{4}{]}^{T}$ and $v_{2}=[r_{1},r_{2},r_{3},r_{4}{]}^{T}$ are constant vectors with respect to $x=[x_{1},x_{2},x_{3},x_{4}{]}^{T}$ the second order Fréchet derivative is


$$F^{\prime \prime }(x)v_{1}v_{2}=\left[\begin{array}{c} \left(h_{3}+h_{4}\right)r_{2}+\left(h_{2}+h_{4}\right)r_{3}+\left(h_{2}+h_{3}\right)r_{4}\\ \mathrm{\backslash noalign}\mathrm{\backslash medskip}\left(h_{3}+h_{4}\right)r_{1}+\left(h_{1}+h_{4}\right)r_{3}+\left(h_{1}+h_{3}\right)r_{4}\\ \mathrm{\backslash noalign}\mathrm{\backslash medskip}\left(h_{2}+h_{4}\right)r_{1}+\left(h_{1}+h_{4}\right)r_{2}+\left(h_{1}+h_{2}\right)r_{4}\\ \mathrm{\backslash noalign}\mathrm{\backslash medskip}h_{2}\;r_{1}+\left(h_{1}+h_{3}\right)r_{2}+\left(h_{2}+h_{4}\right)r_{3}+h_{3}\;r_{4} \end{array}\right]\;,$$

which is a vector of dimension four.

#### 3.1.1. Basin of attraction of proposed method.

**Fig 1:**
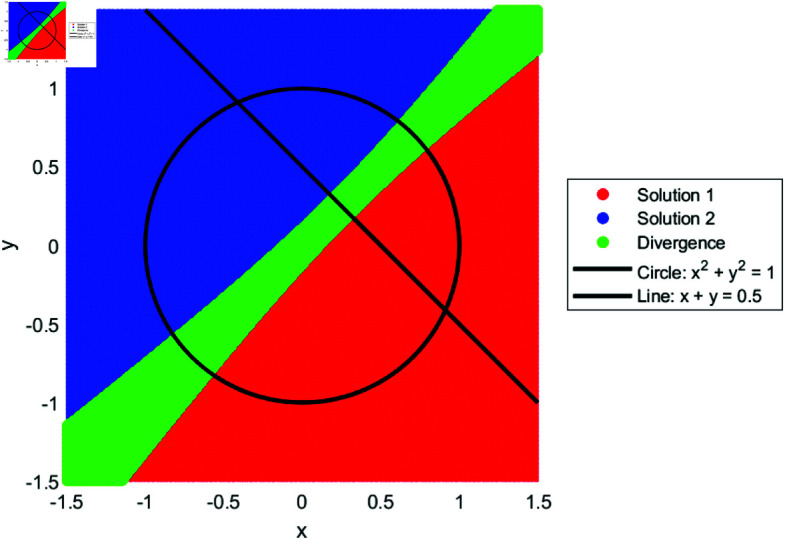
Dynamics of proposed method when a line and circle intersect, the number of iterations are ten and tolerance is 0.01.

**Fig 2:**
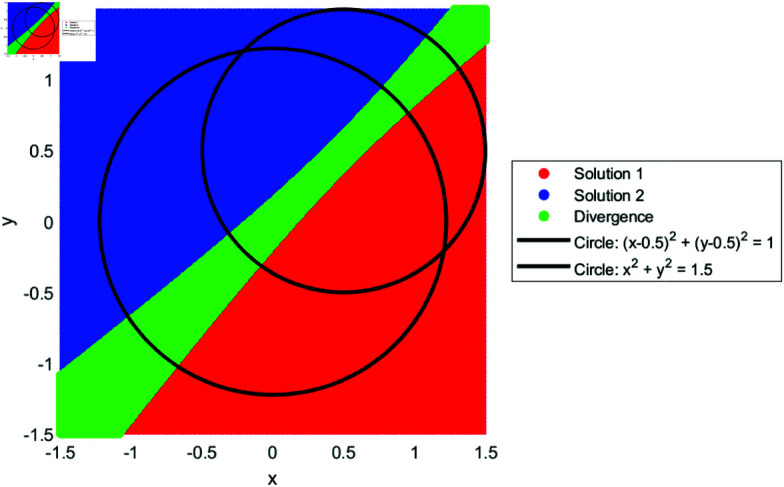
Dynamics of proposed method when a circle and circle intersect, the number of iterations are ten and tolerance is 0.01.

**Fig 3:**
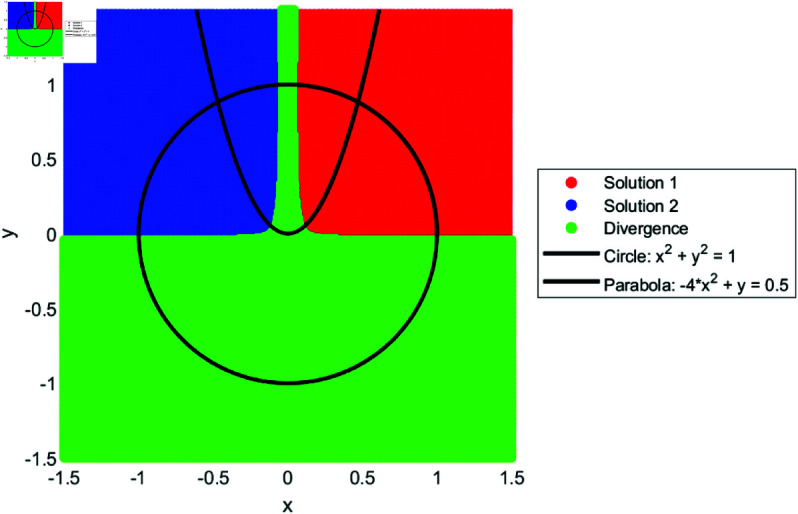
Dynamics of proposed method when a parabola and circle intersect, the number of iterations are ten and tolerance is 0.01.

**Fig 4:**
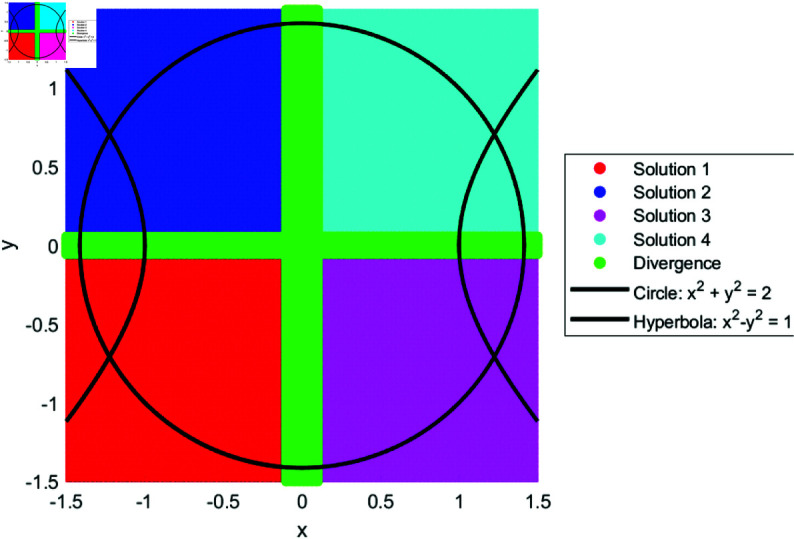
Dynamics of proposed method when a hyperbola and circle intersect, the number of iterations are ten and tolerance is 0.01.

We drew the basin of attraction [14–18] for solutions of SNLEs, assuming these solutions are simple, in order to investigate the dynamics of our suggested numerical iterative technique. For the basin of attraction, we set the parameters for our proposed method as follows: the number of iterations is ten, and the tolerance for the norm of the difference between the current iteration and the known solution is 0.01. The region for the basin of attraction is defined as [−1.5,1.5]×[−1.5,1.5]. In Fig 1, a line and circle intersect in a manner that yields two simple solutions. The red and blue colored regions indicate that initial guesses from these areas converge to the solutions. The green-colored region denotes divergence of the iterative method given the parameter settings. Similarly, Figs 2, 3, and 4 show the basin of attraction for circle to circle, circle to parabola, and circle to hyperbola, respectively. The proposed method demonstrates simple dynamics for the given SNLEs. A Matlab implementation is given in Appendix.

### 3.2 Nonlinear boundary value problems

Here, we show that our proposed numerical scheme is applicable to solve SNLEs associated with nonlinear boundary value problems.

#### 3.2.1 Blasius equation.

The Blasius equation is a nonlinear BVP with quadratic nonlinearity


$$y^{\prime \prime \prime }(x)+\frac{1}{2}y(x)y^{\prime \prime }(x)=0,\;x\epsilon (0,\infty )$$

under the BCs

$$y(0)=0\;,\;y^{\prime }(0)=0\;,\;y^{\prime }(\infty )=1\;.$$

The Blasius equation is defined over a semi-infinite interval [0 , ∞] but it can not simulate the solution over the semi-infinite interval. Assume x _max_ is a large enough number to make the solution asymptotic. To solve the Blasius equation numerically, we must discretize the interval [0,x_max_]. If there are *n* grid points, then $P=\{x_{1},x_{2},\cdots ,x_{n}\}$ and $x_{j}=\frac{x_{\text{max}}}{2}\;(1\;+\;\cos((j\;-\;1)\;\pi \;/(n\;-\;1)))$ for j=1,2,⋯,n. Grid points are not evenly spaced and are denser near the boundaries. The first-order Chebyshev differentiation matrix is *D*_1_, and the higher-order differentiation matrix is computed by multiplying *D*_1_ by *m*, where *m* is the order of differentiation. The rank of *D*_*j*_ is *n − j* If we denote the *j*th order differentiation matrix by $D_{j}=\overset{m-times}{\overbrace{D\times D\times \cdots \times D}}$ . The Chebyshev differentiation matrices perform poorly when the order of differentiation is high, but well when the order of differentiation is low. The matrices are full but offer high accuracy in the numerical approximation of derivatives. The discretized form of Blasius equation is

$$F(𝐲)=B\;𝐲+D_{3}\;𝐲+\frac{1}{2}\;𝐲⊙D_{2}𝐲-𝐪\;,$$ (9)

where 𝐲 is a *n*–dimensional vector and y i = y (xi ) for i =1 ,2 , ⋯ , n. All matrices *B*, *M*_2_, and *M*_3_ are n ×n in size. The operator ⊙ denotes the element-wise multiplication of vectors. The first, second, and last rows of matrices *M*_3_ and *M*_2_ are null. The matrix *B* is a null matrix except for first, second, and third rows. To implement boundary conditions, we create the matrix *B* and the right-hand side vector as


B(1,1)=1

$$B(2,:)=D_{1}(1,:)$$

$$B(n,:)=D_{1}(n,:)$$

q(1)=0;for condition y(x1)=0

q(2)=0;for condition y'(x_1)=0

q(n)=1;for condition y'(x_n)=1.

and q(3:n−1)=0. In all notations, we adopted the Matlab syntax. The computation of higher-order Fréchet derivatives is a necessary part of the implementation of numerical algorithms to solve the SNLEs associated with the discretized Blasius equation.

$$F^{\prime }(𝐲)=B+D_{3}+\frac{1}{2}\left(\text{diag}(D_{2}\;𝐲)+\text{diag}(𝐲)\;M_{2}\right)$$ (10)

$$F^{\prime \prime }(𝐲)\;h_{1}\;h_{2}=\frac{1}{2}\left(h_{1}⊙(D_{2}\;h_{2})+h_{2}⊙(D_{2}\;h_{1})\right)\;.$$ (11)

**Fig 5:**
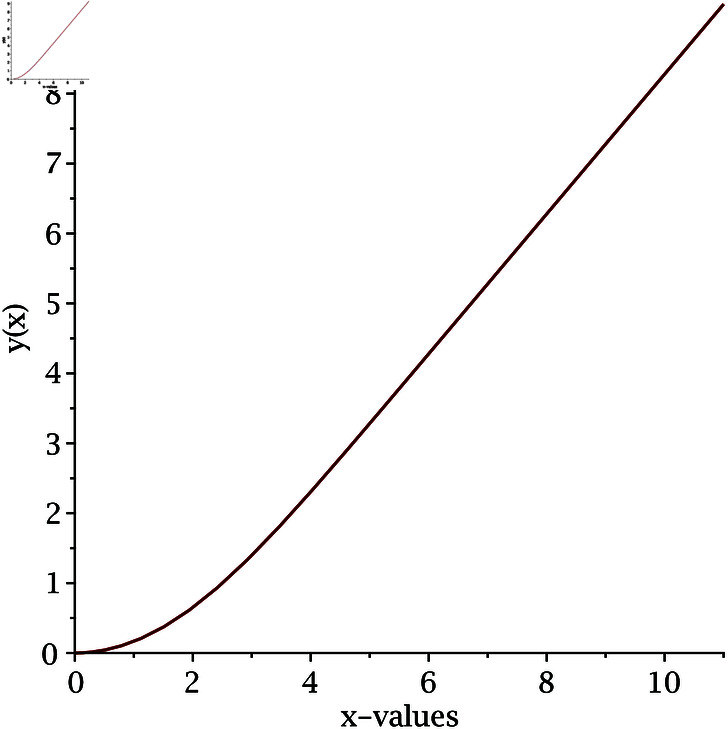
Numerical solution of Blasius equation.

**Fig 6:**
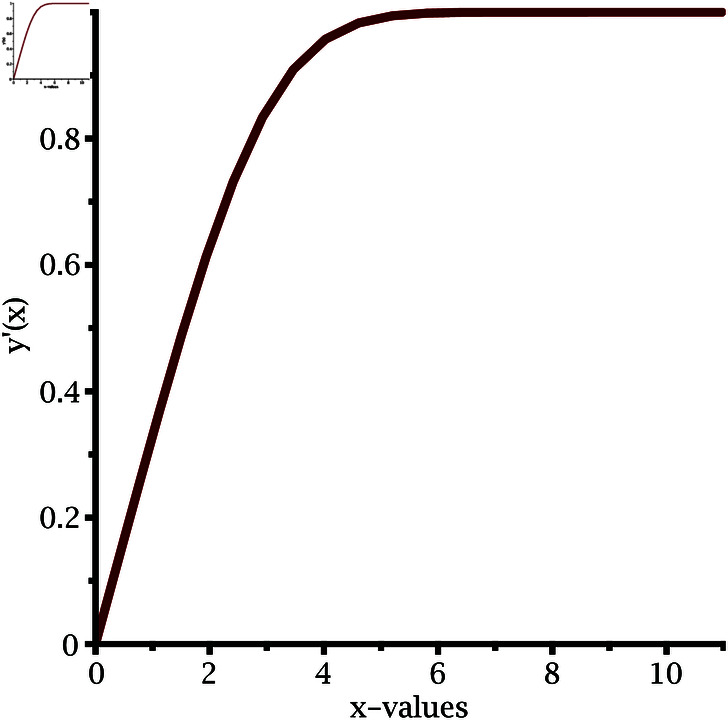
Derivative of numerical solution of Blasius equation.

The implementation of our proposed algorithm is provided in the Appendix. For the numerical approximation of SNLEs associated with the Blasius equation, we take the zero-vector as an initial guess and perform six iterations. Table 2 contains the COC. Our numerical simulations confirm the theoretically claimed CO of our proposed method. The numerical solution of the Blasius equation is plotted along with its derivative in Figs 5 and 6.

**Table 2:** COC of proposed method (2) for the numerical solution of Blasius equation.

Iteration no.	Norm of equations	COC
1	7.697191099	-
2	0.6413099682	-
3	0.01457586112	1.522735257
4	4.710094110e-8	3.340934537
5	3.332832580e-30	4.034203435
6	4.658910519e-119	4.011452022

### 3.3 Falkner–Skan equation

The Blasius boundary layer problem is generalised in Falkner and Skan’s equation [13] by viewing a uniform velocity field *W*_0_ as being divided by a wedge with an angle π β /2. The Falkner–Skan equation is


$$y^{\prime \prime \prime }(x)+\frac{1}{2}y(x)y^{\prime \prime }(x)=0,\;x\epsilon (0,\infty )$$

under the BCs


$$y(0)=0\;,\;y^{\prime }(0)=0\;,\;y^{\prime }(\infty )=1\;.$$

where −0.090429 ≤ β ≤4 /3 is wedge angle. When β=0 the Falkner-Skan equation becomes the Blasius equation. By adopting the notation of Blasius equations, we can write

$$F(y)=B\;y+D_{3}\;y+y⊙D_{2}y+\beta (1-(D_{1}y{)}^{2})-𝐪=0\;,$$ (12)

where B and 𝐪 are identical to those used in Blasius equation. The higher-order Fréchet derivatives can be obtained as

$$F^{\prime }(y)=B+D_{3}+\left(\text{diag}(D_{2}\;y)+\text{diag}(y)\;M_{2}\right)-2\;\beta \;\text{diag}(D_{1}y)\;D_{1}$$ (13)

$$F^{\prime \prime }(y)\;h_{1}\;h_{2}=\left(h_{1}⊙(D_{2}\;h_{2})+h_{2}⊙(D_{2}\;h_{1})\right)-2\;\beta (D_{1}h_{1})⊙(D_{1}h_{2})\;.$$ (14)

**Fig 7:**
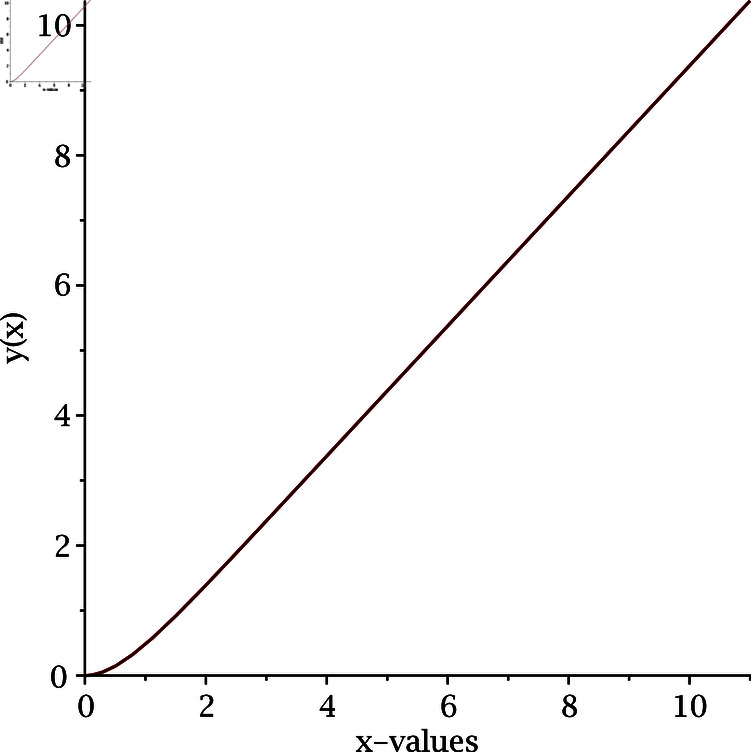
Numerical solution of Falkner-Skan equation for β =4 /3.

**Fig 8:**
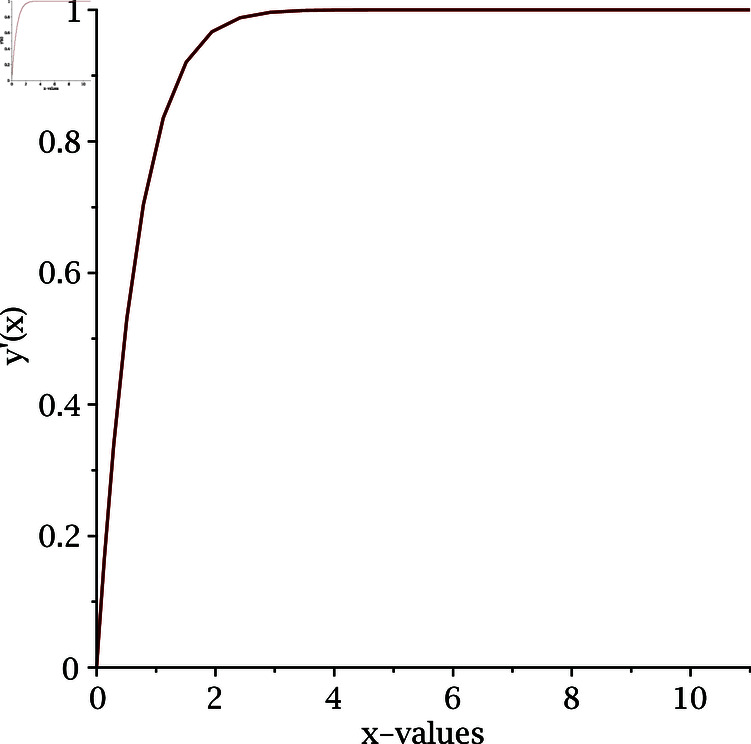
Derivative of numerical solution of Falkner-Skan equation for β =4 /3.

We can observe that the 2^*nd*^-order Fréchet derivative is symmetric, i.e., F ″ (***y*** ) **h1 h2** . The convergence of our proposed method (2) for the numerical approximation of Falkner-Skan equation is depicted in Figs 7 and 8. Whereas, the COC is shown in Table 3.

**Table 3:** COC of proposed method (2) for the numerical approximation of Falkner-Skan equation (β =4 /3).

Iteration no.	Norm of equations	COC
1	1.5e-2	-
2	6.2e-11	-
3	7.3e-44	3.9
4	1.9e-175	4.0
5	1.0e-701	4.0

In the coming subsections, we show that our proposed numerical method 2 is also applicable to solve the nonlinear problem of quadratic nonlinearity in cosmology and computational fluid dynamics.

### 3.4 Lane-Emden equation with index = 2

**Fig 9:**
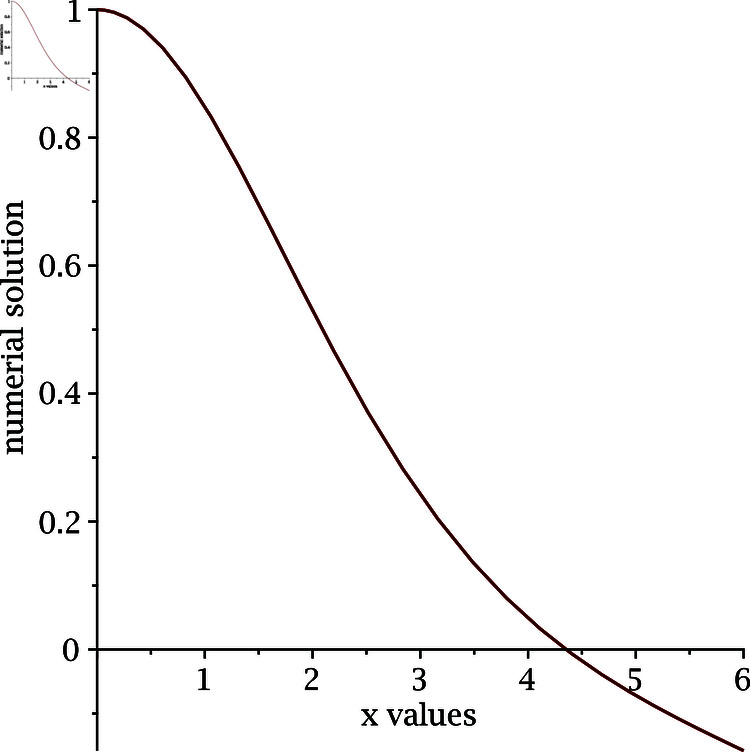
Numerical solution of Lane-Emden equation for n = 2.

**Fig 10:**
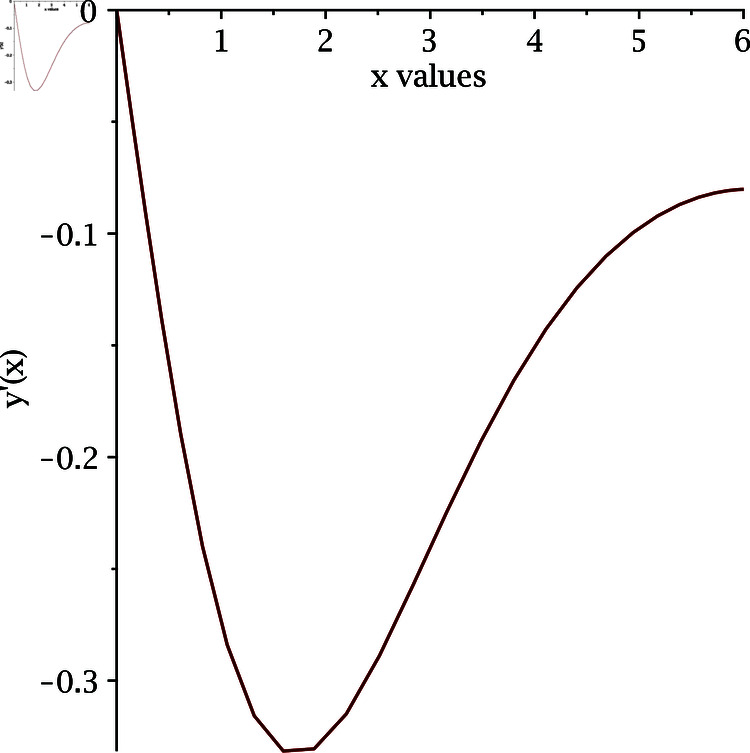
Derivative of numerical solution of Lane-Emden equation for n = 2.

The Lane-Emden equation is a dimensionless Poisson’s equation for polytropic fluid that is spherically symmetric and self-gravitating under the Newtonian gravitational potential. The Lane-Emden equation is

$$\frac{1}{x}\;\frac{d}{dx}\left(x^{2}\;\frac{dy(x)}{dx}\right)+y^{n}=0$$ (15)

under the BCs


$$y(0)=1\;,y^{\prime }(0)=0\;,$$

where *n* is the index of the Lane-Emden equation. As we are dealing with quadratic nonlinearity, we take *n* = 2. Using the numerical iterative method 2, we calculate the numerical solution of the Lane-Emden equation and the results are depicted in Figs 9 and 10. To begin the numerical simulation, the initial guess is y =x, xi ∈ [0 , 6], and y (1 ) =1.

### 3.5 Nano-particles in fluid

A modelling equation for nono-particles in fluid is as follows

$$y^{\prime \prime \prime }‒SA_{1}{1‒\phi }^{2.5}3/2f^{\prime \prime }+x/2f^{\prime \prime }+f^{\prime \prime }f^{\prime \prime }‒ff^{\prime \prime }=0,\;x\in [0,1],$$ (16)

under the BCs


$$f^{\prime }(0)=0\;,f(0)=0\;,f^{\prime }(1)=0\;,f(1)=1/2\;.$$

**Fig 11:**
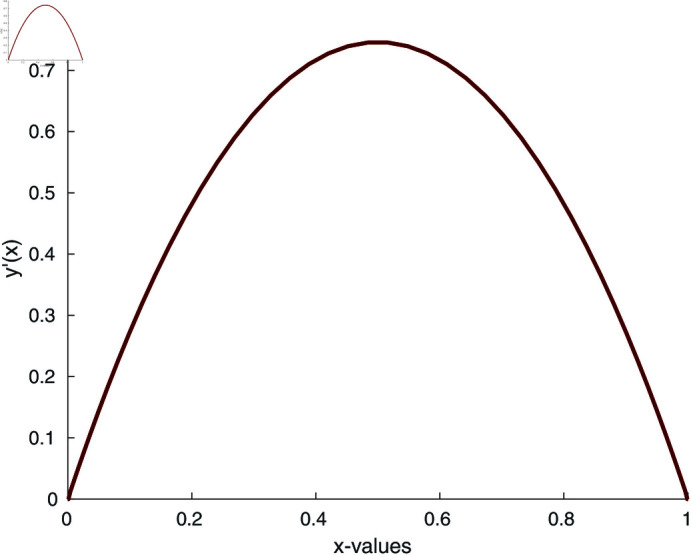
Numerical solution of nano-particles in fluid.

**Fig 12:**
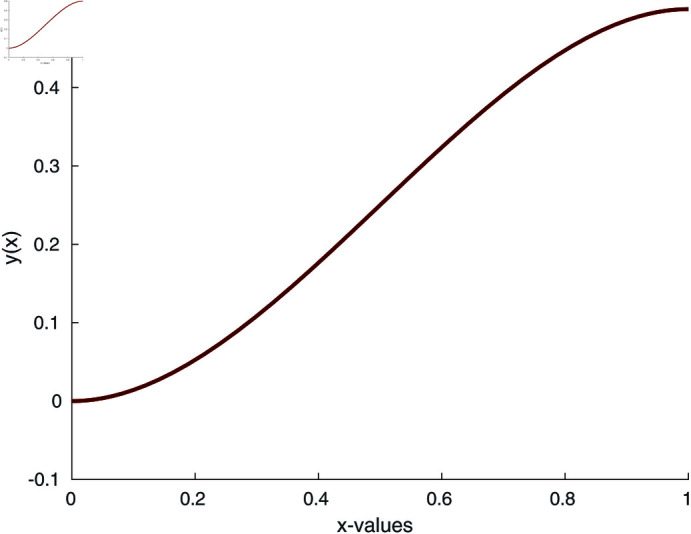
Derivative of numerical solution of nano-particles in fluid.

To simulate the equation (16), we assume the following values of the related parameters:

*k*_*s*_ = 401, *k*_*f*_ = 0.1613, *ρ_s_* = 8933, *ρ*_f_ = 997.1, *c*_*ps*_ = 4179, *S* = 1, ϕ = 0.15, $A_{1}=(1-\phi )+\phi \;(\rho_{s}/\rho_{f})$. The quadratic nonlinearity in (16) is *f′ f″* ‒*f f‴*. The initial guess is a zero vector for the numerical simulation. To compute the numerical solution, the application of our proposed method is depicted in Figs 11 and 12.

### 3.6 Natural convection

A natural convection phenomena can be modelled as

$$A_{1}=(1-\phi )+\phi \;(\rho_{s}/\rho_{f})$$ (17)

$$z^{\prime \prime }+3/4\;\text{Pr}\;y\;z^{\prime }=0\;,$$ (18)

under the BCs


$$y(0)=0\;,y^{\prime }(0)=0\;,y^{\prime }(\infty )=0\;,z(0)=1\;,z(\infty )=0\;.$$

**Fig 13:**
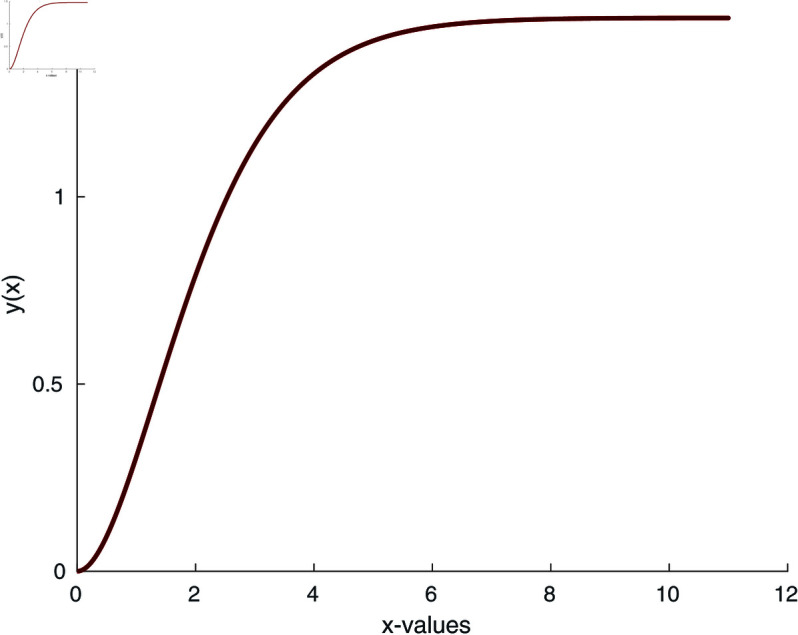
Numerical solution of natural convection (Pr = 1).

**Fig 14:**
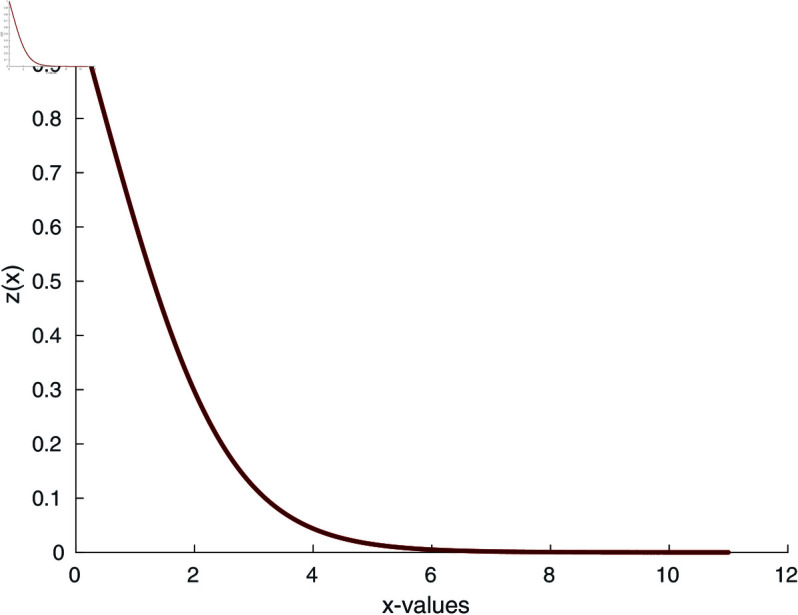
Derivative of numerical solution of natural convection (Pr = 1).

The term $3/4\;y\;y\prime \prime \;-\;(y^{\prime })2/2$ in the preceding equation is quadratically nonlinear. The vector of 1’s is taken as an initial guess for the numerical simulations. For Pr = 1, the numerical solution for the natural convection problem is plotted in Fig 13 and derivative of numerically computed solution is plotted in Fig 14.

### 3.7 Partial differential equations with quadratic nonlinearity with inclusion of semi-linear terms

To test the validity of our proposed algorithm to deal with the quadratic nonlinearity for the boundary value problems in 2-D, we consider the following partial differential equation

$$\alpha (x,y)\;u_{xx}(x,y)+\beta (x,y)\;u_{yy}(x,y)+u^{2}(x,y)=f(x,y)\;,\;\text{where }(x,y)\in (a_{x},b_{x})\times (a_{y},b_{y})\;\text{ with Dirichlet boundary conditions. }$$ (19)

**Fig 15:**
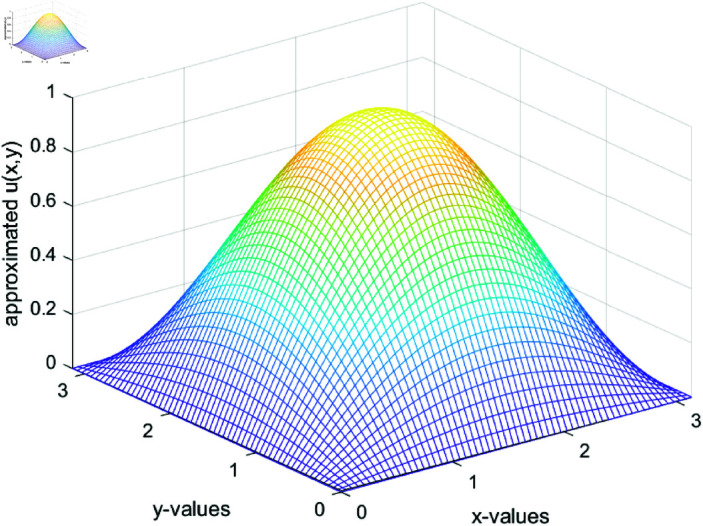
Numerically computing solution of 2-D partial differential equation.

**Fig 16:**
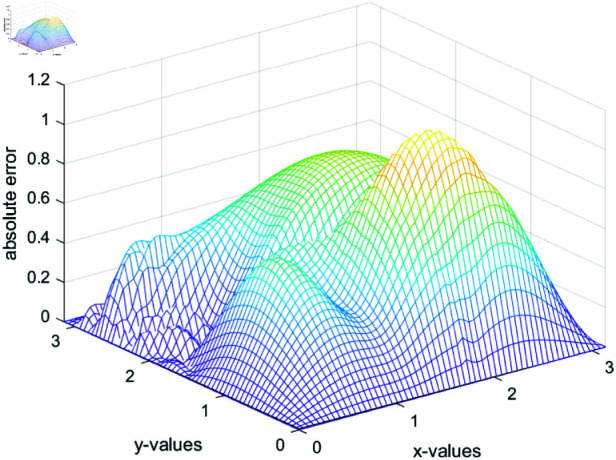
Absolute error in the computed solution of 2-D partial differential equation.

We assume the solution u(x,y)=sin(x)sin(y)and(x,y)∈(0,π)2. One can compute the right hand term f(x,y)=−(α(x,y)+β(x,y))u(x,y)+u(x,y)2. To perform the simulations, we choose α(x,y)=sin(x+y)~and~β(x,y)=cos(x+y). To make comparison of Newton and our propose method, we ran twenty simulations of each method and results are depicted in Table 4. The Table 4 shows that our proposed numerical iterative method is **1.7118** times faster than the classical Newton method. The numerically computed solution of 2-D partial differential equation and the absolute errors are shown in Figs 15 and 16, respectively. Matlab code of our proposed method for the solution of partial differential equation is provided in the appendix.

**Table 4:** Average simulation elapsed times for the comparison of Newton and our proposed method.

	Newton Method	Proposed Method
Problem type	2-D Nonlinear boundary value problem	same
Size of system of nonlinear equations	60×60	same
No. of simulations for time evaluation	20	same
Criteria for the convergence	1.0 ×10^−10^	same
Average simulation elapse time	0.59163	0.34562

## 4 Conclusions

In the present research, we focused on the BVPs that have quadratic nonlinearity. There is a large class of physics problems that have quadratic nonlinearity by definition. To get the benefit of the quadratic nonlinearity, we developed a single-point numerical iterative method that uses the information of the 2^*nd*^-order Fréchet derivative. Our proposed method assumed that the 2^*nd*^-order Fréchet derivative should be constant, and hence all the higher-order Fréchet derivatives are zero tensors. The efficiency index of our method is higher than that of the Newton method because the Newton method attains CO 2 by using two function evaluations, whereas our method attains CO 4 with the same number of function evaluations. The Newton method needs to solve a single system of linear equations, while our method uses three systems of linear equations. To make the proposed method efficient, we calculate the LU factors of the Jacobian and then solve three lower and upper triangular systems. In all test examples, we have proved the validity of our proposed method. In all cases, the COC is almost four, which is aligned with the theoretically approved CO of our proposed method. Our proposed method is not valid when the nonlinearity is not quadratic.

## Appendix

**Maple implementation of our proposed method for the Blasius equation.**

> restart;

> Digits := 1000;

> with(LinearAlgebra);

> n := 30;

> n := n - 1;

> x := Vector(n + 1, i -> cos(1.0*Pi*(i - 1)/n));

> c1 := Vector(n + 1, 1);

> c1[1] := 2.0;

> c1[n + 1] := 2.0;

> c2 := Vector(n + 1, i -> (-1)^(i - 1));

> c := Vector(n + 1, i -> c1[i]*c2[i]);

> X := Matrix(n + 1, n + 1, (j, i) -> x(j));

> XT := Matrix(n + 1, n + 1, (i, j) -> x(j));

> dX := X - XT;

> A := dX + 1.0*IdentityMatrix(n + 1);

> B := Matrix(n + 1, n + 1, (i, j) -> c(i)/(c(j)*A(i, j)));

> U := DiagonalMatrix(Vector(n + 1, i -> add(B[i, j],

j::integer = 1 .. n + 1)));

> M := U - B;

> x := Vector(n + 1, i -> x[n + 2 - i]);

> a := 0;

> b := 11;

> x := 0.5*‘+‘ ((b - a)*x, a + b);

> M1 := 2*M/(b - a);

> M2 := M1 . M1;

> M3 := M2 . M1;

> B := Matrix(n + 1);

> B[1, 1] := 1;

> B[2, 1 .. n + 1] := M1[1, 1 .. n + 1];

> B[n + 1, 1 .. n + 1] := M1[n + 1, 1 .. n + 1];

> M2[[1, 2, n + 1], 1 .. n + 1] := 0;

> M3[[1, 2, n + 1], 1 .. n + 1] := 0;

> Rhs := Vector(n + 1);

> Rhs[n + 1] := 1;

> y := Vector(n + 1);

> iter := 6;

> normf := Vector(iter + 1);

> M2y := M2 . y;

> f := (B . y) + (M3 . y) + 0.5*(y .  M2y) - Rhs;



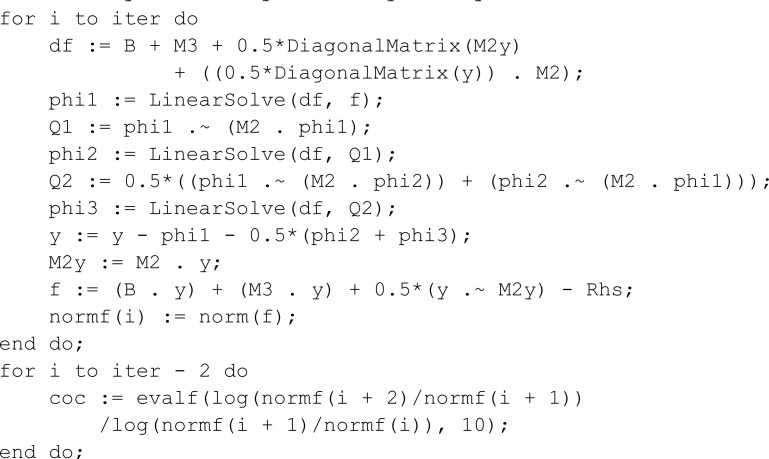



**Matlab implementation of basin of attraction for our proposed method.**

clc; clear all; close all;

% Define the four solutions to the system of equations

s1 = [-1.224745; -0.707107];

s2 = [-1.224745; 0.707107];

s3 = [1.224745; -0.707107];

s4 = [1.224745; 0.707107];

% Number of iterations for the proposed method and

tolerance for convergence

iter = 10;

tolerance = 0.01;

% Generate a grid of initial guesses for the proposed method

x_range = linspace(-1.5, 1.5, 400);

y_range = linspace(-1.5, 1.5, 400);

[X, Y] = meshgrid(x_range, y_range);

% Initialize a matrix to store the convergence status of

each initial guess

status = zeros(size(X));

% 0 indicates non-convergence,

%1-4 indicate convergence to one of the four solutions

% Loop over all initial guesses

for ix = 1:numel(X)

% Set the current initial guess

x = [X(ix); Y(ix)];

% Perform the iterative proposed  method



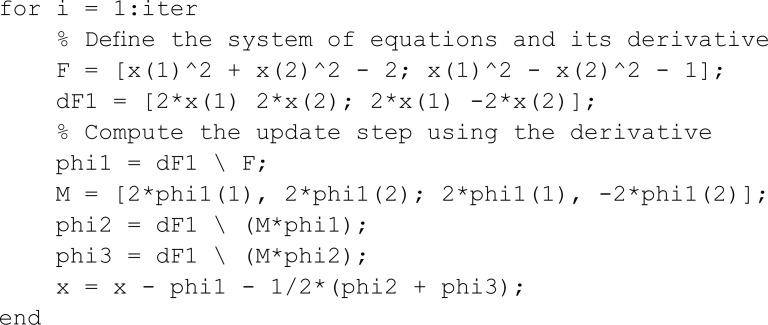



% Check for convergence to one of the solutions

F = [x(1)^2 + x(2)^2 - 2; x(1)^2 - x(2)^2 - 1];



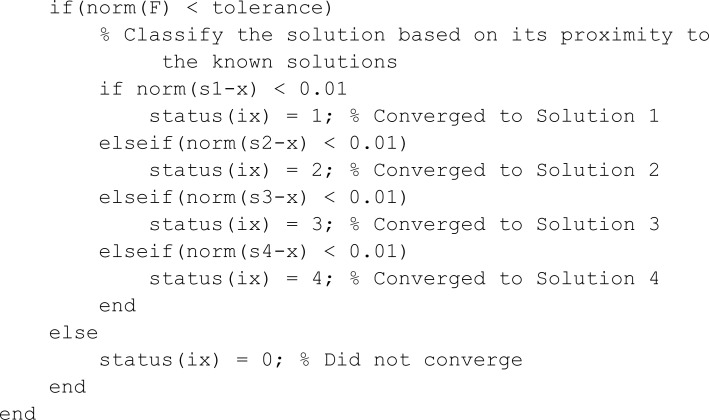



% Plotting the results

figure; hold on;

% Plot points corresponding to each of the four solutions

in different colors

scatter(X(status == 1), Y(status == 1), 1, ’r’, ’filled’,...

’DisplayName’, ’Solution 1’);

scatter(X(status == 2), Y(status == 2), 1, ’b’, ’filled’,...

’DisplayName’, ’Solution 2’);

scatter(X(status == 3), Y(status == 3), 1, ’m’, ’filled’,...

’DisplayName’, ’Solution 3’);

scatter(X(status == 4), Y(status == 4), 1, ’c’, ’filled’,...

’DisplayName’, ’Solution 4’);

% Plot divergent points with a distinct marker

scatter(X(status == 0), Y(status == 0), 25, ’g’, ’filled’,...

’DisplayName’, ’Divergence’);

% Draw the circle and hyperbola defined by the system of equations

fimplicit(@(x,y) x.^2 + y.^2 - 2, [-1.5 1.5 -1.5 1.5],

’LineWidth’, 2,...

’Color’,’k’, ’DisplayName’, ’Circle: x^2 + y^2 = 2’);

fimplicit(@(x,y) x.^2 - y.^2 - 1, [-1.5 1.5 -1.5 1.5],

’LineWidth’, 2,...

’Color’,’k’, ’DisplayName’, ’Hyperbola: x^2-y^2 = 1’);

% Label axes, show the legend, and set other plot properties

xlabel(’x’);

ylabel(’y’);

legend(’Location’, ’eastoutside’);

legend show;

axis equal;

grid on;

xlim([-1.5, 1.5]);

ylim([-1.5, 1.5]);

hold off;

**Matlab implementation of proposed method for solving nonlinear partial differential equation**

clc;

clear all;

close all;

format short g

% Define the partial differential equation

% alpha(x,y) * u_xx + beta(x,y) * u_yy + u^2 = f(x,y)

% The exact solution is u(x,y) = sin(x) * sin(y)

% The function f(x,y) is derived from this exact solution.

% Define the exact solution function

u = @(x,y) sin(x).*sin(y);

% Define coefficient functions alpha(x,y) and beta(x,y)

alpha = @(x,y) sin(x + y);

beta  = @(x,y) cos(x + y);

% Define the source term f(x,y)

f = @(x,y)  -(alpha(x,y) + beta(x,y)) * u(x,y) + u(x,y)^2;

% Define domain boundaries

ax = 0; bx = pi;

ay = 0; by = pi;

% Define number of grid points in x and y directions

nx = 60;

ny = 60;

% Indexing function to convert 2D index (i, j) to 1D index

eta = @(i,j) j + (i - 1) * ny;

% Define grid step sizes in x and y directions

hx = (bx - ax) / (nx - 1);

hy = (by - ay) / (ny - 1);

% Define grid points in x and y

x = (ax:hx:bx)’;

y = (ay:hy:by)’;

% Initialize matrices and vectors

n = nx * ny;

A = zeros(n); % Matrix for linear system

B = eye(n);   % Identity matrix for Newton’s method

fvec = zeros(n, 1); % Source term vector

umat = zeros(nx, ny); % Initial guess matrix

% Loop through grid points to build matrix A and vector fvec

k = 1;

for i = 1:nx

for j = 1:ny

if (i == 1 || i == nx || j == 1 || j == ny)

% Boundary conditions: Dirichlet boundary conditions

A(k,k)  = 1;

fvec(k) = u(x(i), y(j));

B(k,k) = 0;

else

% Fill matrix A for interior points using finite difference

A(k, eta(i-1,j)) = alpha(x(i), y(j)) / hx^2;

A(k, eta(i,j-1)) = beta(x(i), y(j)) / hy^2;

A(k, eta(i,j))   = -2 * (alpha(x(i), y(j)) / hx^2 + beta(x(i),

y(j)) / hy^2);

A(k, eta(i,j+1)) = beta(x(i), y(j)) / hy^2;

A(k, eta(i+1,j)) = alpha(x(i), y(j)) / hx^2;

% Fill the source term vector fvec

fvec(k) = f(x(i), y(j));

end

k = k + 1;

% Store the exact solution for later comparison

umat(i,j) = u(x(i), y(j));

end

end

% Remove small values (numerical noise) from A and fvec

A(abs(A) < 1.e-14) = 0;

fvec(abs(fvec) < 1.e-14) = 0;

% Newton’s method parameters

iter = 20;   % Maximum number of iterations

tol = 1.0e-10;  % Convergence tolerance

diagB = diag(B); % Diagonal of matrix B

% Timing setup for performance evaluation

time = 0;  % Initialize total time

REPS = 10;  % Number of repetitions for averaging time

% Loop for performance evaluation over multiple runs

for j = 1:REPS

tstart = tic;  % Start timing for this repetition

U = umat(:); % Initial guess as a vector

% Newton’s method loop

for i = 1:iter

% Compute the residual vector F

F = A * U + diagB .* (U.^2) - fvec;

norm_F = norm(F); % Compute the norm of the residual

% Check for convergence

if (norm_F < tol)

disp(’success’);

iterations = i;

break;

end

% Compute the Jacobian matrix dF

dF = A + 2 * diag(diagB .* U);

Z = decomposition(dF, ’lu’);  % LU decomposition

% Solve for phi1, phi2, and phi3 using LU decomposition

phi1 = Z F;

phi2 = Z (2 * diagB .* (phi1.^2));

phi3 = Z (2 * diagB .* (phi1 .* phi2));

% Update the solution vector U

U = U - phi1 - 0.5 * (phi2 + phi3);

end

telapsed = toc(tstart);  % Time taken for this repetition

time = time + telapsed;  % Accumulate total time

end

% Compute and display the average time over all repetitions

average_time = time / REPS;

disp([’Average time: ’, num2str(average_time)]);

% Reshape the solution vector U back to a matrix

W = reshape(U, nx, ny);

% Plot the approximated solution

figure

mesh(x, y, W’);

xlabel(’x-values’)

ylabel(’y-values’)

zlabel(’approximated u(x,y)’)

% Plot the absolute error between the exact and approximated

solutions

figure

mesh(x, y, abs(umat - W)’);

xlabel(’x-values’)

ylabel(’y-values’)

zlabel(’absolute error’)
